# O-52 - A MULTI-COUNTRY OUTBREAK OF HEPATITIS A ASSOCIATED WITH CONSUMPTION OF A FROZEN MIXED BERRY PRODUCT, UNITED KINGDOM AUGUST 2024 – MARCH 2025

**DOI:** 10.1093/pubmed/fdag046.046

**Published:** 2026-07-09

**Authors:** Ms Caitlin McCarthy, Tatiana Garcia Vilaplana, Saba Savul, Louisa Ellis, Alison Smith-Palmer, Lesley Larkin, Caoimhe McKerr, David Leeman, Koye Balogun, Miranda Mindlin, Siew Lin Ngui, Rory Gunson, Samantha Shepherd, Andrew Nelson, Danielle McMichael, Patrick McAleavey, Tina Potter, Agnieszka Bird, Laura Lafferty, Christina Anthony

**Affiliations:** Public Health Scotland; UK Health Security Agency; Public Health Scotland; Public Health Scotland; Public Health Scotland; Public Health Scotland; Public Health Scotland; UK Health Security Agency; UK Health Security Agency; UK Health Security Agency; UK Health Security Agency; West of Scotland Specialist Virology Centre; West of Scotland Specialist Virology Centre; Public Health Wales; Public Health Agency Northern Ireland; Public Health Agency Northern Ireland; Food Standards Agency; Food Standards Agency; Food Standards Scotland; Food Standards Scotland

## Abstract

**Background:**

Public Health Scotland (PHS) and UK Health Security Agency (UKHSA) were notified by national reference laboratories of multiple hepatitis A virus (HAV) genotype IB isolates of UKHSA sequence VRD25_HAV017.

**Objectives:**

Investigations characterised VRD25_HAV017 UK cases, assessed potential sources and informed public health actions.

**Methods:**

Cross-UK collaboration enabled aligned surveillance questionnaires and timely data sharing. International communications commenced via EpiPulse.

Confirmed UK hepatitis A cases are routinely sequenced. VRD25_HAV017 underwent sequence comparison using HAVNet and UKHSA reference databases.

Case data were collected using surveillance questionnaires as standard. Investigations included i) descriptive epidemiology, ii) food traceback investigation on strong descriptive signals, iii) retrospective case-case study.

Study case-controls were HAV cases with non-VRD25_HAV017 sequences. Cases and case-controls were primary non-travel-associated cases resident in the UK with completed food histories and specimen dates between August 2024-March 2025. A multivariable model was developed using Firth’s logistic regression, including a-priori confounders and food exposure variables with p<0.2 in the univariable analysis.

**Results:**

Twenty-two primary cases(22/23) occurred between August 2024-March 2025. Cases were distributed nationally, travelled infrequently(3/22), and frequently reported frozen berry consumption(any 20/22; mixed-retailer A 14/19; named product A 7/13). Sequence VRD25_HAV017 clustered with UK travellers returning from Slovakia (UKHSA) and sequences reported by Slovakia in 2025 (HAVNet). Outbreak cases were strongly associated with reporting consumption of product A (aOR:69, 95%CIs:3-15402, p=0.005).

Food traceback identified one product manufacturer in Europe, sourcing berries from growers in four countries.

Another European country identified matching cases reporting consumption of mixed frozen berry product B with 4/6 shared berry types, but a different manufacturer in the same country. A retained batch tested positive for hepatitis A (not sequenced).

**Conclusions:**

Combined descriptive epidemiological, analytical, sequencing and traceback findings, alongside parallels with the European investigation, indicate frozen mixed berry product A as the likely infection source.

Descriptive epidemiological evidence can be sufficient to initiate early food traceback.

